# Do not keep it simple: recent advances in the generation of complex organoids

**DOI:** 10.1007/s00702-020-02198-8

**Published:** 2020-05-08

**Authors:** Philipp Wörsdörfer, Takashi I, Izumi Asahina, Yoshinori Sumita, Süleyman Ergün

**Affiliations:** 1 Institute of Anatomy and Cell Biology, University of Würzburg, Würzburg, Germany; 2 Department of Regenerative Oral Surgery, Unit of Translational Medicine, Nagasaki University, Nagasaki, Japan; 3 Basic and Translational Research Center for Hard Tissue Disease, Nagasaki University, Nagasaki, Japan

**Keywords:** Organoid, Stroma, Vasculature, Neural, Microglia, Blood vessel

## Abstract

3D cell culture models which closely resemble real human tissues are of high interest for disease modelling, drug screening as well as a deeper understanding of human developmental biology. Such structures are termed organoids. Within the last years, several human organoid models were described. These are usually stem cell derived, arise by self-organization, mimic mechanisms of normal tissue development, show typical organ morphogenesis and recapitulate at least some organ specific functions. Many tissues have been reproduced in vitro such as gut, liver, lung, kidney and brain. The resulting entities can be either derived from an adult stem cell population, or generated from pluripotent stem cells using a specific differentiation protocol. However, many organoid models only recapitulate the organs parenchyma but are devoid of stromal components such as blood vessels, connective tissue and inflammatory cells. Recent studies show that the incorporation of endothelial and mesenchymal cells into organoids improved their maturation and might be required to create fully functional micro-tissues, which will allow deeper insights into human embryogenesis as well as disease development and progression. In this review article, we will summarize and discuss recent works trying to incorporate stromal components into organoids, with a special focus on neural organoid models.

## Introduction

During embryonic development, complex tissues and organs arise by self-organization. This process involves the interaction of different tissue compartments, e.g. mesenchyme, epithelium, and blood vessels. Cell–cell interaction and multilineage communication among different cells via cytokines trigger the full maturation of tissues finally enabling organ specific function.

Pluripotent stem cell-based organoid cultures are state of the art in vitro platforms recapitulating fundamental aspects of organogenesis, which allow researchers to model and investigate human development and diseases. Moreover, they represent promising tools for drug discovery and toxicity testing as well as studies on irradiation effects. However, many organoids appear incomplete as they lack stromal components such as blood vessels, connective tissue, peripheral nerves, and immune cells. Recent studies on liver organoids suggest that intercellular signaling between mesenchymal cells, endothelial cells, and hepatocytes is required for proper organoid maturation and it is likely that similar interactions play a role in other tissues and organ systems as well. For that reason, an incorporation of stromal components into the already existing organoid models may improve their function and bring these models one step closer to the original tissue architecture and physiological function. Furthermore, such complex organoids could help to reduce the number of animal experiments in the future.

In this short review article, we will summarize the recent works trying to incorporate stromal components into organoids, with a special focus on neural organoid models.

## Organoids

Cells in our body permanently interact with other cell types or extracellular matrix components. This interaction can be mediated by direct cell–cell or cell–matrix contacts or secreted factors. During embryonic development, this environment controls processes such as cellular differentiation, maturation, migration, polarization or morphogenesis and creates physiological niches for stem cells. Self-organization finally results in complex tissues. In a similar way, diseases also evolve in a tissue context. For that reason, many mechanisms driving embryonic development but also the origin of disease cannot be properly addressed in vitro in 2D cell cultures. This underlines the need for more realistic 3D in vitro tissue models, the so-called organoids.

The observation that single cell suspensions made from primary embryonic tissues have the remarkable ability to reaggregate and self-organize into tissue structures which in many aspects closely resemble the original tissue is not new. Early reports describe the reconstitution of tissues (Moscona and Moscona 1952) and even organ-like structures (Weiss and Taylor 1960) from single-cell suspensions of chick embryos in vitro. Regarding the brain, reaggregation and histogenesis of fetal mouse isocortex and hippocampus has been already studied in 1970 (Delong 1970). However, the identification and isolation of specific adult stem cell populations, such as Lgr5 + intestinal stem cells (Sato et al. 2009), which have the ability to continuously regrow their specific epithelium with all its cell types in 3D cell culture is a rather new finding that had a strong impact especially on the stem cell field and opened up a world of new possibilities for different areas of scientific research (Huch et al. 2017).

Within the last few years, several human organoid models were developed. These are usually stem cell derived, mimic mechanisms of normal tissue development, show typical organ morphogenesis, and recapitulate at least some organ specific functions. Many tissues have been reproduced in the lab such as gut (Sato et al. 2009; Spence et al. 2011), brain (Lancaster et al. 2013), lung (Dye et al. 2015), kidney (Takasato et al. 2016), and liver (Takebe et al. 2013). The resulting entities can be either derived from an adult stem cell population, e.g. primary Lgr5 + intestinal stem cells (Sato et al. 2009) or generated from pluripotent stem cells (PSCs) using a specific differentiation protocol (Spence et al. 2011; Mithal et al. 2020).

Primary stem cell-derived organoids are interesting screening platforms for personalized medicine. To establish these models, human patient material, e.g. gut or brain tissue, is essentially required. The access is usually granted during surgical removal of tumor tissue such as intestinal cancer or glioblastoma. From the primary material, individual organoids can be rapidly established and utilized for patient-specific treatment strategies (van de Wetering et al. 2015; Jacob et al. 2020).

A second, more commonly used approach, is pluripotent stem cell (PSC)-derived organoids. Human PSCs were first established in 1998 and are originally derived from the inner cell mass of a blastocyst-stage embryo (Thomson et al. 1998). The need to sacrifice human pre-implantation embryos led to an ethical debate regarding the use of human PSCs, the so-called embryonic stem cells, and to legal regulations varying between different countries. Nowadays, induced pluripotent stem cells (iPSCs) mostly substitute for the controversially debated embryo-derived PSCs, solving many ethical issues (Takahashi and Yamanaka 2006). iPSCs can be generated from different terminally differentiated human cell types such as skin fibroblasts (Takahashi et al. 2007), mononucleated peripheral blood cells (Staerk et al. 2010) or urine-derived kidney epithelial cells (Zhou et al. 2011; Bharadwaj et al. 2013). During this process, their epigenome is altered in a way that it corresponds to the epigenome of a pluripotent stem cell. This is usually achieved by the overexpression of a certain set of reprogramming factors (e.g. Oct4.c-Myc, Sox2, and Klf4). Once the reprogramming progress is completed, the cells no longer depend on the transgenes, but intrinsically maintain the pluripotent state due to their new epigenetic program/signature.

iPSCs can be grown for many passages in cell culture and easily manipulated with modern gene editing tools such as CRISPR/Cas9 (Jinek et al. 2012; Mali et al. 2013). This allows for the generation of different iPS cell lines with disease-specific genetic mutations or gene knockouts. Moreover, iPSCs can be directly derived from patients affected by genetic disorders.

## Most organoids lack a stromal compartment

The majority of organoid models are generated by inducing and culturing a 3D cell cluster of either primary or iPS cell-derived epithelial stem cells (e.g. neural organoids, gastrointestinal organoids, lung organoids etc.). From these stem cells, all cell types of the corresponding epithelium arise and form the parenchymal organ compartment by self-organization. Moreover, stem cell niches are created preserving a pool of undifferentiated cells. A good example are gut organoids which are derived from Lgr5^+^ stem cells of the intestinal epithelium (Sato et al. 2009) (Mithal et al. 2020) or cerebral organoids originating from Sox1^+^ neuroepithelial stem cells (Lancaster et al. 2013). As such organoids derive from an ectodermal (brain) or endodermal (gut) epithelial progenitor, they exclusively consist of epithelial cells or cell types derived thereof. For that reason, they stay inherently incomplete as stromal components are lacking. The stroma is the framework of connective tissue within organs plus the including blood vessels, lymph vessels, immune cells, and peripheral nerves. Most parts of the stroma arise from the mesoderm. For that reason, in contrast to the above-mentioned examples, kidney organoids, which derive from a mesodermal progenitor, contain stromal compartments and a rudimentary vascular network because angioblasts are concurrently induced with nephron progenitor cells (Homan et al. 2019). Of note, not all stromal cells originate from the mesoderm. The head mesenchyme, for example, arises from the neural crest, an ectodermal cell population delaminating from the neural plate border during neural tube formation and closure.

## Why we should not keep it simple

Conventional organoids remain incomplete as they lack stromal components. But is this really of importance? Recent studies investigating liver organoids come to an interesting conclusion. The authors revealed that liver organoids get more mature and functional if the hepatoblasts are co-cultured with mesenchymal stem cells and endothelial cells (Asai et al. 2017; Ayabe et al. 2018; Camp et al. 2017; Goulart et al. 2019). They recognized a multilineage communication between endothelial cells, mesenchymal cells, and the hepatoblasts. Mesenchymal cells and hepatoblasts release vascular endothelial growth factor (VEGF) which stimulates endothelial network formation (Camp et al. 2017). On the other hand, mesenchymal cells and endothelial cells modulate TGFβ and Wnt signaling that controls the hepatoblast fate (Ayabe et al. 2018; Goulart et al. 2019). The incorporation of non-parenchymal cell types finally resulted in an increased albumin production and liver-specific enzyme expression suggesting higher tissue maturation (Goulart et al. 2019; Camp et al. 2017; Ayabe et al. 2018). A similar observation was made after the incorporation of mesenchymal stem cells into lung organoids which improved alveolar differentiation (Leeman et al. 2019). These results demonstrate that multilineage communication drives tissue maturation, an effect that is very likely not restricted to liver organoids but rather represents a common principle.

## Neural organoid models

In the following paragraphs, we will set the focus mostly on neural organoids. These aggregates of brain tissue are usually derived from pluripotent stem cells (PSCs). For the generation of neural organoids, PSCs are grown in suspension culture where they aggregate to form cell clusters. Cells within the resulting aggregates can be further induced to adopt a neuroepithelial phenotype by using a neuro-inductive cell culture medium. This finally results in neuroepithelial spheres consisting of Sox1^+^ precursor cells (Lancaster et al. 2013). The neuroepithelial aggregates are embedded into an extracellular matrix (e.g. Matrigel or basement membrane extract (BME)) and are further cultivated in 3D suspension for tissue maturation. Neural organoids consist of neuroepithelial stem cell compartments lining ventricle-like cavities and different neural cell types derived thereof (radial glia cells, neurons, astrocytes, oligodendrocytes, ependymal cells, etc.). In the case of cerebral organoids, a radial migration of neurons originating from the stem cell zone can be observed and a multi-layered cortex-like tissue organization is established upon organoid maturation recapitulating the aspects of brain cortex development (Lancaster et al. 2013). Because organoids mimic embryonic and fetal development in real-time, it can take more than 100 days until embryonically late cell types such as astrocytes or oligodendrocytes arise and complete the artificial brain tissue (Madhavan et al. 2018; Marton et al. 2019; Sloan et al. 2017; Pasca et al. 2015). A targeted and timely controlled manipulation of specific signaling pathways (e.g. Wnt signaling, TGFβ signaling, BMP signaling, SHH signaling etc.) during neural induction can be further applied to induce brain region-specific organoids (Jo et al. 2016; Qian et al. 2016; Xiang et al. 2019). As neural organoids are generated from neuroectodermal progenitor cells, they lack a vascular system as well as brain-specific macrophages, the so-called microglia.

Neural organoids are fascinating tools to investigate human neural development in vitro. Moreover, they rise hopes to understand the mechanism of diseases affecting the human brain or brain development (Tian et al. 2020). However, many relevant human diseases affecting the nervous system are late-onset diseases, e.g. Parkinson’s or Alzheimer’s disease, which usually start to manifest at an age of approximately 60 years. It could be difficult to fully address such diseases in an organoid model and might require strategies for accelerated maturation such as progerin-induced aging (Miller et al. 2013).

## Recent strategies for the incorporation of stromal compartments into organoids

Several recent studies addressed the issue of increasing organoid complexity by incorporation of stromal components. Most of these studies focused on the establishment of a vascular system. The following paragraph will summarize recent developments in the field in more detail. First, we will address the aspects of organoid vascularization in general. In a separate paragraph, we will set a special focus on neural organoid vascularization.

One of the first studies concerning organoid vascularization was published by Takebe and co-workers (2015). The authors demonstrated that self-condensation of endothelial cells, mesenchymal cells, and specific parenchymal cell types results in the formation of vascularized organ buds. When such structures were transplanted into a mouse, the vasculature got connected to the host circulation and was blood perfused. This protocol was initially developed with murine cells but could be also transferred to the human system (Takahashi et al. 2018). Instead of being assembled from a mixture of terminally differentiated cell types (endothelial cells, smooth muscle cells, and mesenchymal stem cells), multi-layered human blood vessels can also arise via self-organization from iPS cell-derived mesodermal progenitor cells (MPCs) (Worsdorfer et al. 2019; Wimmer et al. 2019). These are induced by activating Wnt and BMP signaling (Patsch et al. 2015). If MPCs are grown as 3D aggregates in suspension culture or if they are directly induced from 3D spheres of undifferentiated iPSCs, they spontaneously differentiate and self-organize forming a vascularized mesenchymal aggregate, a so-called blood vessel organoid (Wimmer et al. 2019). Such organoids can be transplanted into mice to yield a fully functional blood vessel system and may serve as a human platform to model vascular diseases such as diabetic vasculopathy.

## Recent strategies for neural organoid vascularization

Several approaches to achieve neural organoid vascularization have been explored within the last few years (Fig. 2). The first study co-cultured cerebral organoids with iPS cell-derived endothelial cells and transplanted the resulting vascularized organoid into the brain of a mouse (Pham et al. 2018). Another study mixed ETS variant 2 (ETV2) overexpressing iPS cells with unaltered normal iPS cells, grew the mixture as aggregates in suspension culture, and induced neural differentiation (Cakir et al. 2019). ETV2 is a transcription factor that is essential and sufficient to convert cells into an endothelial cell fate (Sumanas and Lin 2006) (Morita et al. 2015). For that reason, ETV2 overexpressing iPSCs turn into endothelial cells even under neurogenic conditions. A different approach uses transplantation of cerebral organoids into the brain of a host organism e.g. a mouse (Mansour et al. 2018; Daviaud et al. 2018). This finally results in the functional vascularization of the transplanted human tissue by mouse vessels. The strategies presented above result at least in neural organoids with a network of endothelial tubes which can be blood perfused. However, a blood vessel is more complex than an endothelial tube. Larger vessels are constructed by three different layers. The intimal endothelial layer, the medial smooth muscle compartment as well as the adventitial connective tissue that was shown to harbor non-vascular and vascular progenitors that contribute to new vessel formation (Mekala et al. 2018; Worsdorfer et al. 2017; Klein et al. 2014). Even small capillaries are enwrapped by pericytes. Therefore, incorporation of endothelial cells into neural organoids is not enough to generate bona fide blood vessels. As mentioned above, mesenchymal organoids which harbor a functional network of multi-layered blood vessels can be grown from mesodermal progenitor cells (Wimmer et al. 2019; Worsdorfer et al. 2019). We recently demonstrated that co-culturing mesenchymal and neural organoids results in partially vascularized neural organoids (Worsdorfer et al. 2019). We think that using mesodermal progenitor cells, which can deliver endothelial cells, smooth muscle cells, pericytes as well as mesenchymal stem cells might be the preferred way to induce blood vessels either in organoids or biofabricated or engineered tissue constructs.

Of note, neural organoids with an endothelial network can be also derived by using primary material from brain surgery. A good example is primary glioblastoma organoids which reflect the cellular heterogeneity of the tumor in cell culture and often preserve the vascular network of the original tissue (Jacob et al. 2020). Moreover, microglia can be also found in this type of organoid. Such organoids are useful to explore patient-specific treatment strategies. However, due to the limited access to the patient material, PSC-derived organoids are more commonly used. Moreover, PSC-derived organoids enable to research neurodevelopmental aspects.

## Incorporation of microglia into neural organoids

**Fig. 1:**
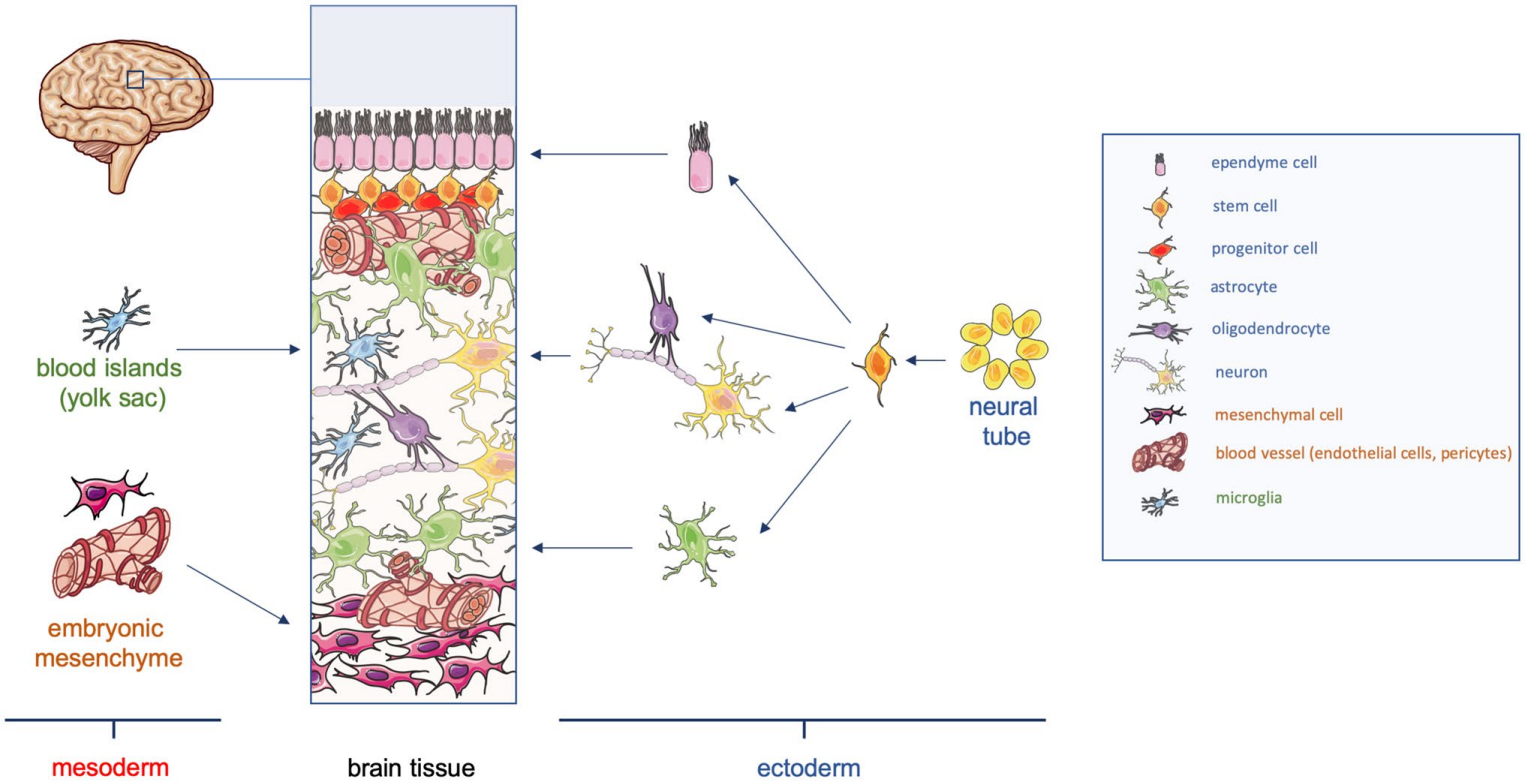
Cellular composition of the nervous tissue. The nervous tissue consists of neurons and glial cells. Among the glial cells, astrocytes, oligodendrocytes, microglia, and ependymal cells can be found. Moreover, a stem and progenitor niche exists in close association with the ependymal cells as well as brain blood vessels. The surface of the brain tissue is covered by a layer of connective tissue including blood vessels, forming the meninges. While neurons, astrocytes, oligodendrocytes, and ependymal cells arise from a common Sox1^+^ neuroepithelial stem cell, the blood vessels and the meningeal connective tissue derive from the embryonic mesenchyme. In contrast, microglia are generated early during development in the yolk sac blood islands and subsequently infiltrate the nervous tissue to become brain tissue resident, self-maintaining macrophages. This schematic is composed of graphical elements taken from the image bank from Servier Medical Art licensed under a Creative Commons Attribution 3.0 Unported License (https://creativecommons.org/licenses/by/3.0)

**Fig. 2:**
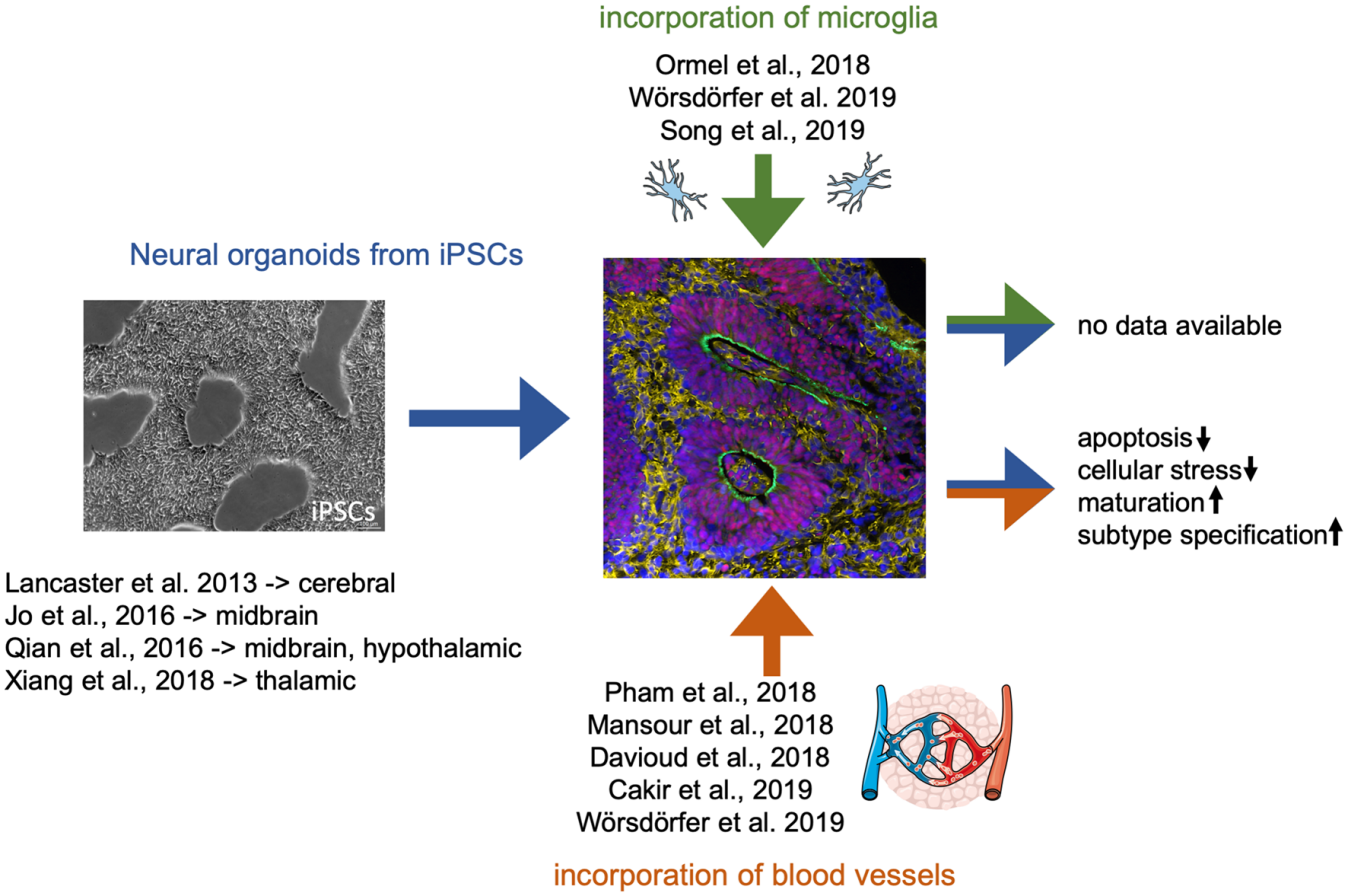
Incorporation of microglia and blood vessels into neural organoids. This fig. shows recent publications describing (1) the generation of brain region specific neural organoids (Jo et al. 2016; Qian et al. 2016; Xiang et al. 2019), (2) the incorporation of microglia into neural organoids and (3) the incorporation of a vascular system into neural organoids as well as the so far demonstrated advantages of increasing organoid complexity. The phase contrast image (left side) shows undifferentiated iPSCs in 2D culture. The immunofluorescence image (right side) shows an immature neural organoid generated from hiPSCs stained for Sox1 (neural stem cells—> red), MAP2 (neurons—> yellow), and N-Cadherin (green). Graphical elements within this schematic were taken from the image bank from Servier Medical Art licensed under a Creative Commons Attribution 3.0 Unported License (https://creativecommons.org/licenses/by/3.0)

Besides blood vessels and connective tissue, most organoids currently published also lack immune cells. Among these, tissue resident macrophages are of high importance as they are discussed to be involved in the developmental processes and morphogenesis (Wynn et al. 2013; Mekala et al. 2018). Moreover, they could become important players if it comes to disease modeling in organoids. Macrophages derive from monocytes which are generated in the bone marrow. They reside within the circulation for approximately 1 day until they infiltrate the interstitial space to become macrophages. Tissue resident macrophages have a different origin and are thought to derive from early sites of embryonic hematopoiesis e.g. the yolk sac (Perdiguero et al. 2015). They infiltrate the embryonic tissues early during development and maintain themselves throughout life time (Stremmel et al. 2018). In brain tissue, these macrophages are called microglia (Fig. 1). As microglia, like all blood cells, derive from the mesodermal lineage, conventional cerebral organoids which are grown from neural stem cells do not contain this cell type. Two recent studies address this issue (Fig. 2). They show that the incorporation of mesodermal progenitor cells into neural organoids results in the formation of blood islands from angioblasts (Ormel et al. 2018; Worsdorfer et al. 2019). These structures give rise to macrophages which migrate from the mesenchymal compartment into the neural part of the organoid. Here they adopt a microglia-like phenotype switching from an amoeboid to a ramified morphology and show a microglia-specific gene expression profile. Moreover, they are able to do phagocytosis. The number of macrophages originating from mesodermal blood islands could be further boosted and more precisely controlled by adding stimulating cytokines, such as interleukin 7 (IL7), stem cell factor (SCF), insulin-like growth factor 2 (IGF2), and fibroblast growth factor 2 (FGF2), as recently described for the generation of hematopoietic organoids (Motazedian et al. 2020). Other recent studies use a protocol for the differentiation of microglia from iPS cells (Muffat et al. 2016) and describe their co-culture with different types of neural organoids (Song et al. 2019; Muffat et al. 2018). Incorporation of microglia into human brain organoids can be further achieved by transplanting organoids into a mouse brain (Mansour et al. 2018). However, in this case, microglia are of mouse origin and infiltrate the transplanted tissue coming from adjacent brain regions. Finally, microglia are also found in organoids derived from primary human brain tissue such as glioblastoma organoids (Jacob et al. 2020).

So far, there is no data on the effect of microglia on neural organoid maturation yet. However, this is an important and interesting topic to be investigated in future studies.

## Why do we need complex neural organoids?

Several recent studies present promising strategies to implement a vascular system as well as microglia-like cells into neural organoids either by incorporating endothelial cells (Pham et al. 2018) or mesodermal progenitors (Worsdorfer et al. 2019) or by transplantation into the brain of a mouse (Mansour et al. 2018; Daviaud et al. 2018) (Fig. 2). But do we really need the vascular/stromal compartment?

During development, the vascular system and the brain, although generated by two different germ layers, develop simultaneously. For that reason, it is not surprising that blood vessels and neural cell types are discussed to instruct each other to finally shape the nervous tissue. Indeed, early during nervous system development, the so-called perineural vascular plexus forms triggered by VEGF release from progenitor cells within the neural tube (Hogan et al. 2004). At later stages, other factors such as Wnt7a (Stenman et al. 2008; Daneman et al. 2009) acting via GPR124 (Anderson et al. 2011; Cullen et al. 2011; Kuhnert et al. 2010) and TGFβ (Siqueira et al. 2018) are released by cells of the nervous system controlling brain blood vessel development. On the other hand, blood vessels are essential components of the neural stem cell niche within the brain (Fig. 1) (Shen et al. 2004; Culver et al. 2013) and soluble factors released by endothelial cells (Wnt components, VEGF, CXCL12, semaphorins, pleiotrophin) as well as direct cell–cell contacts either preserve neural stem cell identity or induce their differentiation. Finally, the brain vasculature can serve as guide structure for newborn neurons or axons (for excellent review see Paredes et al. (2018)).

The first study that compares neurons in vascularized versus non-vascularized cerebral organoids found that the incorporation of a vascular system accelerates functional maturation of neurons as determined by single cell RNA sequencing (Cakir et al. 2019). Besides a possible lack/delay in functional maturation, conventional organoids without vascular system are limited in size and develop apoptotic core regions because of insufficient supply of the inner cells with nutrients and oxygen (Lancaster et al. 2013) This can be frequently observed in cerebral organoids as soon as they exceed a diameter of approximately 500 µm and is accompanied by metabolic cell stress leading to a reduced number of cellular subtypes as compared to normal fetal brain tissue (Bhaduri et al. 2020). Interestingly, transplantation into a mouse brain decreased apoptosis (Mansour et al. 2018), reduced stress markers, and increased cellular subtype specification (Bhaduri et al. 2020). Therefore, to reduce apoptosis, avoid cellular stress, and drive functional maturation, a functional blood vessel network as found in almost any tissue in vivo is essentially required. Recent studies on vascularized cerebral organoids in vitro show that even if those blood vessels are not connected to a circulatory system, the extent of cell death decreases and functional maturation of neurons is accelerated (Cakir et al. 2019).

## Conclusion

Organoid models are valuable state of the art in vitro platforms for the investigation of human developmental processes as well as mechanisms of disease. However, these model systems are still in their infancy as they often lack important components of the real organs, in particular connective tissue, blood vessels, and inflammatory cells. Moreover, they are devoid of a peripheral nervous system. These structures are of importance if organoids shall be used to model diseases associated with processes such as fibrosis, vascular remodeling or inflammation. Moreover, conventional organoids do not reach complete functional maturation as well as cellular subtype specification. They are limited in size and stressed due to an insufficient supply with oxygen and nutrients.

Important advances have been made in the last few years to introduce the above-mentioned stromal components into the already-established organoid models. Important challenges for future studies will be to (1) develop robust and highly reproducible protocols for the generation of organoids with connective tissue, functional vasculature as well as inflammatory cells and (2) decipher interlineage communication pathways and their impact on tissue maturation in detail e.g. by using single-cell Omics. To further increase complexity, developmental potential, and functionality, different types of organoids could be assembled into larger structures e.g. by combining vascularized mesenchymal organoids with neural organoids (Worsdorfer et al. 2019), different brain-region specific organoids with each other (Xiang et al. 2017, 2019; Birey et al. 2017) or organoids from different regions of the gut (Koike et al. 2019). All these add-ons will help to engineer more realistic tissue models in vitro.
